# Uncovering Gender Dimensions in Antimicrobial Resistance: A 10-Year Study of Clinical Bacterial Isolates in Uganda

**DOI:** 10.7759/cureus.93762

**Published:** 2025-10-03

**Authors:** Joel Bazira, Nakato Constance Nakimuli, Nalumaga Pauline Petra, Brenda Nakazibwe, Abel W Walekhwa, Kawuma Simon, Hope Derick, Iramiot Jacob, Lawrence Mugisha

**Affiliations:** 1 Microbiology and Parasitology, Mbarara University of Science and Technology, Mbarara, UGA; 2 Microbiology, Mbarara University of Science and Technology, Mbarara, UGA; 3 Epidemiology and Public Health, IDEMU Mathematical Modeling Unit, Kampala, UGA; 4 Veterinary Medicine, University of Cambridge, England, GBR; 5 Computer Science, Mbarara University of Science and Technology, Mbarara, UGA; 6 Department of Medical Laboratory Sciences, Muni University, Arua, UGA; 7 Department of Microbiology and Immunology, Busitema University, Mbale, UGA; 8 Infectious Diseases, College of Veterinary Medicine, Animal Resources, and Bio-Security, Makerere University, Kampala, UGA

**Keywords:** amr surveillance, antimicrobial resistance, gender disparities, global health, multidrug resistance, uganda.

## Abstract

Background: Antimicrobial resistance (AMR) and multidrug resistance (MDR) are escalating global health threats, particularly in low- and middle-income countries (LMICs). Understanding gender-specific resistance patterns is essential for inclusive antimicrobial stewardship and gender-targeted interventions.

Objective: This study aimed to investigate gender-specific trends in AMR and MDR among clinical bacterial isolates collected from Mbarara Regional Referral Hospital, Uganda, within a 10-year period (2014-2024).

Methods: A total of 4,170 non-duplicate clinical isolates subjected to antimicrobial susceptibility testing (AST) were retrospectively analyzed. Gender-specific resistance patterns were calculated and compared using the Wilcoxon signed-rank test, Chi-square, and Fisher’s exact test. All analyses were performed in Python (Google Colab).

Results: Among all isolates, 92.7% were resistant to at least one antibiotic, while 71.6% were multidrug resistant. While resistance appeared higher in female-submitted isolates in unadjusted analysis, adjusted models showed that urinary AMR was significantly higher among reproductive-age women (OR = 1.38, 95% CI: 1.22-1.57), while non-urine AMR was elevated in male-submitted specimens from children (OR = 1.45, 95% CI: 1.20-1.76) and adults aged ≥50 (OR = 1.36, 95% CI: 1.13-1.63). MDR showed distinct organism-gender associations: it was more common in male-associated urinary *Enterobacterales* (OR = 1.41, 95% CI: 1.10-1.82), in female-linked non-urine *Staphylococcus aureus* (OR = 1.62, 95% CI: 1.16-2.27), and in male-linked *Pseudomonas aeruginosa* (OR = 1.56, 95% CI: 1.12-2.18).

Conclusion: This study reveals distinct gender disparities in AMR and MDR patterns, structured by age, specimen type, and organism. These findings support the integration of gender-sensitive variables such as pregnancy status, contraceptive use, and care-seeking behavior to better explain resistance pathways and support gender-responsive AMR control in LMICs.

## Introduction

Antimicrobial resistance (AMR) is one of the most pressing public health challenges of the 21st century, contributing to approximately 1.27 million deaths and disproportionately affecting low- and middle-income countries (LMICs) where diagnostic capacity and antimicrobial stewardship infrastructure remain limited [1]. In Uganda and across Sub-Saharan Africa, AMR has significantly contributed to mortality associated with bloodstream and urinary tract infections, particularly those caused by *Escherichia coli* and *Klebsiella pneumoniae* [2,3].

Multidrug resistance (MDR), defined as resistance to at least one agent in three or more antimicrobial classes, exacerbates the challenge by narrowing therapeutic options and increasing treatment failures, costs, and mortality [4]. While surveillance systems increasingly monitor AMR trends by bacterial species and drug class, few studies have examined resistance trends through a gender lens, especially in LMIC settings.

Biological, behavioral, and structural factors contribute to sex-based differences in infection risk and antibiotic exposure. Women, for instance, experience more frequent urinary tract infections and are more likely to receive empiric antibiotic treatment for reproductive or urogenital symptoms, potentially leading to greater antimicrobial exposure and resistance development [5,6]. Despite this, gender remains a largely neglected variable in microbiological surveillance systems and AMR research in Africa [7].

Evidence from high-income settings has demonstrated sex-based AMR differences, for example, higher aminopenicillin resistance in women and higher methicillin resistance in men [8]. However, comparable analyses from sub-Saharan Africa are scarce. Systematic reviews have highlighted the urgent need for research exploring the intersection of gender and AMR in African contexts, where health system inequities may amplify underlying disparities [6]. Therefore, this study aimed to determine gender-specific trends in AMR and MDR and to generate evidence to inform equitable surveillance strategies and gender-sensitive antimicrobial stewardship efforts.

## Materials and methods

Study area

This was a retrospective, cross-sectional study conducted at Mbarara Regional Referral Hospital (MRRH) in southwestern Uganda. MRRH is a tertiary-level hospital approximately 260 kilometers from Kampala and serves as a teaching hospital for Mbarara University of Science and Technology. The hospital caters to multiple departments, including internal medicine, obstetrics and gynecology, pediatrics, surgery, oncology, and emergency care, drawing referrals from neighboring districts and bordering countries (Rwanda and Tanzania) [9].

Study period and data source

Clinical microbiology data were retrieved for the period January 2014 to December 2024. Laboratory records were extracted from the computerized database of the microbiology unit at MRRH. 

Sample size and inclusion criteria

A total of 4,170 non-duplicate clinical bacterial isolates from various specimen types were analyzed.

Inclusion criteria were as follows: availability of complete AST data, clear identification of patient sex, non-duplicate isolate per patient per infection episode, and clinical relevance of the sample source. Only bacterial isolates with complete antimicrobial susceptibility testing (AST) profiles and recorded patient sex were included.

The specimens were collected from a broad range of clinical sites, such as urine, pus, blood, sputum, tracheal aspirate, wound swab, pleural fluid, and ascitic tap.

Culture methods and susceptibility testing

The collected samples were inoculated with a 0.01-mL loop on blood, chocolate, and MacConkey agar (HiMedia, India) and incubated at 37 °C for 24 hours. Detected pathogens in significant amounts were identified according to phenotypical characteristics (colonial characteristics on the culture media, biochemical tests such as Gram staining, catalase, oxidase, triple sugar iron, and sulfur indole motility) [10]. AST was performed using the Kirby-Bauer disk diffusion method on Mueller-Hinton agar and interpreted according to Clinical and Laboratory Standards Institute (CLSI) guidelines.

Bacterial pathogens under investigation in this study were *Staphylococcus aureus, Escherichia coli, Klebsiella pneumoniae, Klebsiella *species*, Streptococcus pneumoniae, Salmonella *species*, Citrobacter species, Pseudomonas aeruginosa, Pseudomonas *species*, Enterobacter *species*, Acinetobacter *species*, Enterococcus *species, and *Streptococcus *species. Organism-antibiotic combinations with at least 30 isolates only were investigated to ensure statistical reliability and minimize sampling bias.

Antibiotics tested were grouped according to pharmacologic class. These included penicillins (amoxicillin, amoxicillin/clavulanic acid, ampicillin, penicillin, oxacillin, and piperacillin), cephalosporins (cefaclor, cefazolin, cefepime, cefixime, cefotaxime, cefovecin, cefoxitin, ceftriaxone, and cefuroxime), carbapenems (imipenem), monobactams (aztreonam), aminoglycosides (amikacin, gentamicin), fluoroquinolones (ciprofloxacin, levofloxacin), macrolides (azithromycin, erythromycin), lincosamides (clindamycin), tetracyclines (doxycycline, tetracycline), sulfonamides (sulfamethoxazole), phenicols (chloramphenicol), oxazolidinones (linezolid), glycopeptides (vancomycin), and nitrofurans (nitrofurantoin). Control strains used were *S. aureus* ATCC 25923 and *K. pneumoniae* ATCC 700603.

AMR was defined as resistance against a single or multiple antibiotics [11]. Multidrug resistance (MDR) was defined as resistance against single or multiple agents in three or more antibiotic classes [12]. Gender-based analysis used the total number of isolates tested for every bacterium-antibiotic combination as the denominator.

Data analysis

Data were analyzed in Python 3.10 (Google Colab), using individual bacterial isolates as the unit of analysis. Resistance was defined as the proportion of resistant (R) plus intermediate (I) outcomes relative to the total tested (R+I+S). Descriptive epidemiology quantified sex distribution and sex-specific prevalence, with denominators explicitly defined for overall isolates, resistant isolates, and MDR isolates. Visual summaries included bar charts of sex and MDR share, and heatmaps of resistance by organism and antibiotic class. Cells with fewer than 30 isolates were masked.

Inferential analyses compared male-female differences at the organism-antibiotic level for 99 prespecified pairs using chi-square tests, with overall direction summarized by a Wilcoxon signed-rank test. Resistance was modeled separately for urine and non-urine specimens using robust binomial GLMs with logit link, frequency weights, and adjustment for age. MDR was modeled at the isolate level with sex, age band, specimen, organism, and patient department. Final models were selected by cross-validation and refit on the full dataset. Results are reported as adjusted and stratified odds ratios, where values >1 indicate higher resistance or MDR among female isolates.

## Results

Distribution of clinical specimens by gender

A total of 4,170 clinical specimens for which AST was performed between 2014 and 2024 were analyzed in this study. These were from 2,726 patients, of which 1,544/2,726 (56.6%) were female patients and 1,182/2,726 (43.4%) were male patients. The most common specimens were urine samples, which accounted for over one-third of all specimens, with 852 (62.6%) from female patients and 509 (37.4%) from male patients, as shown in Table 1.

**Table 1:** Gender distribution of clinical specimens.

Specimen type	Female n(%)	Male n(%)	Total (n)
Ascitic tap	11(52.4)	10(47.6)	21
Blood culture	417 (46.6)	478 (53.4)	895
Ear swab	45 (47.4)	50 (52.6)	95
High vaginal swab (HVS)	518 (100)	0	518
Myocardial infarct tissue	47 (62.7)	28 (37.3)	75
Pleural fluid	13 (39.4)	20 (60.6)	33
Pus swab	190 (50.4)	187 (49.6)	377
Sputum	228 (44.4)	285(55.6)	513
Stool	15 (53.6)	13 (46.4)	28
Throat swab	29 (51.8)	27 (48.2)	56
Tracheal aspirate	28 (35.4)	51 (64.6)	79
Urine	852 (62.6)	509 (37.4)	1,361
Wound swab	68 (57.1)	51 (42.9)	119
Total	2461 (59.0)	1709 (41.0)	4,170

Distribution of antibiotic-resistant isolates by gender

**Figure 1:**
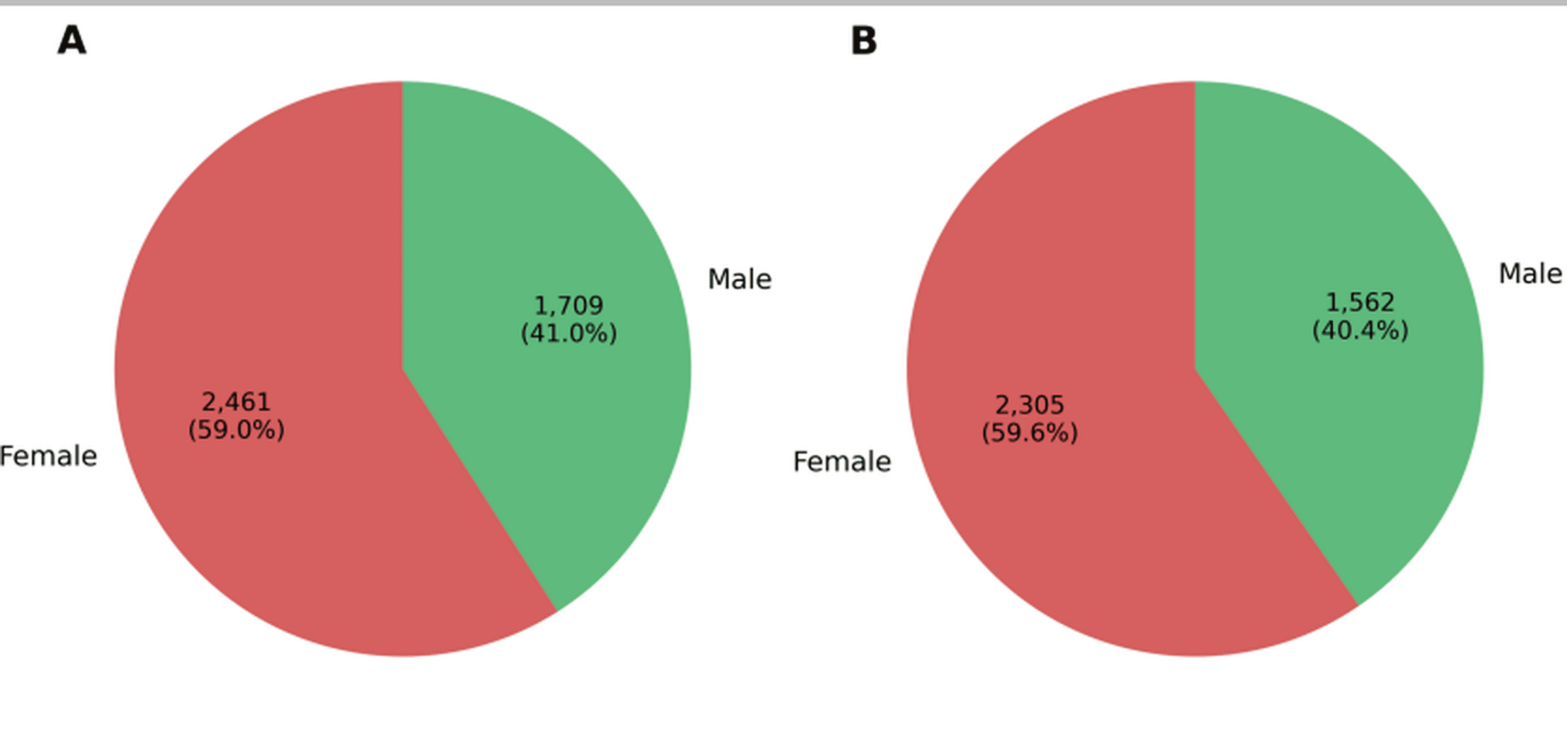
Sex distribution of bacterial isolates. Panel A shows the proportion of isolates recovered from female and male patients across all cultures. Panel B shows the corresponding distribution among isolates that were resistant to at least one antibiotic.

A total of 2,461 (59.0%) came from female patients and 1,709 (41.0%) from male patients. Overall, 3,867 of 4,170 (92.7%) isolates were resistant to one or more antibiotics. Among resistant isolates, 2,305 (59.6%) were from females and 1,562 (40.4%) from males (Figure 1). 

**Figure 2:**
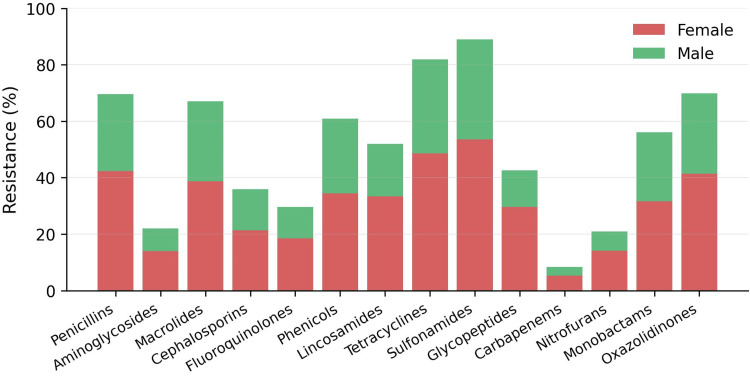
Antibiotic resistance by gender and drug class. Distribution of bacterial isolates by organism and patient sex. Bars represent the proportion of isolates identified from cultures of samples collected from female and male patients for each organism. Percentages are displayed to indicate relative contribution within each organism.

Resistance to penicillins, macrolides, tetracyclines, and sulfonamides was higher in female isolates, while resistance to aminoglycosides, glycopeptides, and fluoroquinolones showed relatively smaller differences between genders, while Carbapenem resistance remained lower in both groups (Figure 2).

Antibiotic resistance patterns among Gram-positive and Gram-negative bacteria

**Figure 3:**
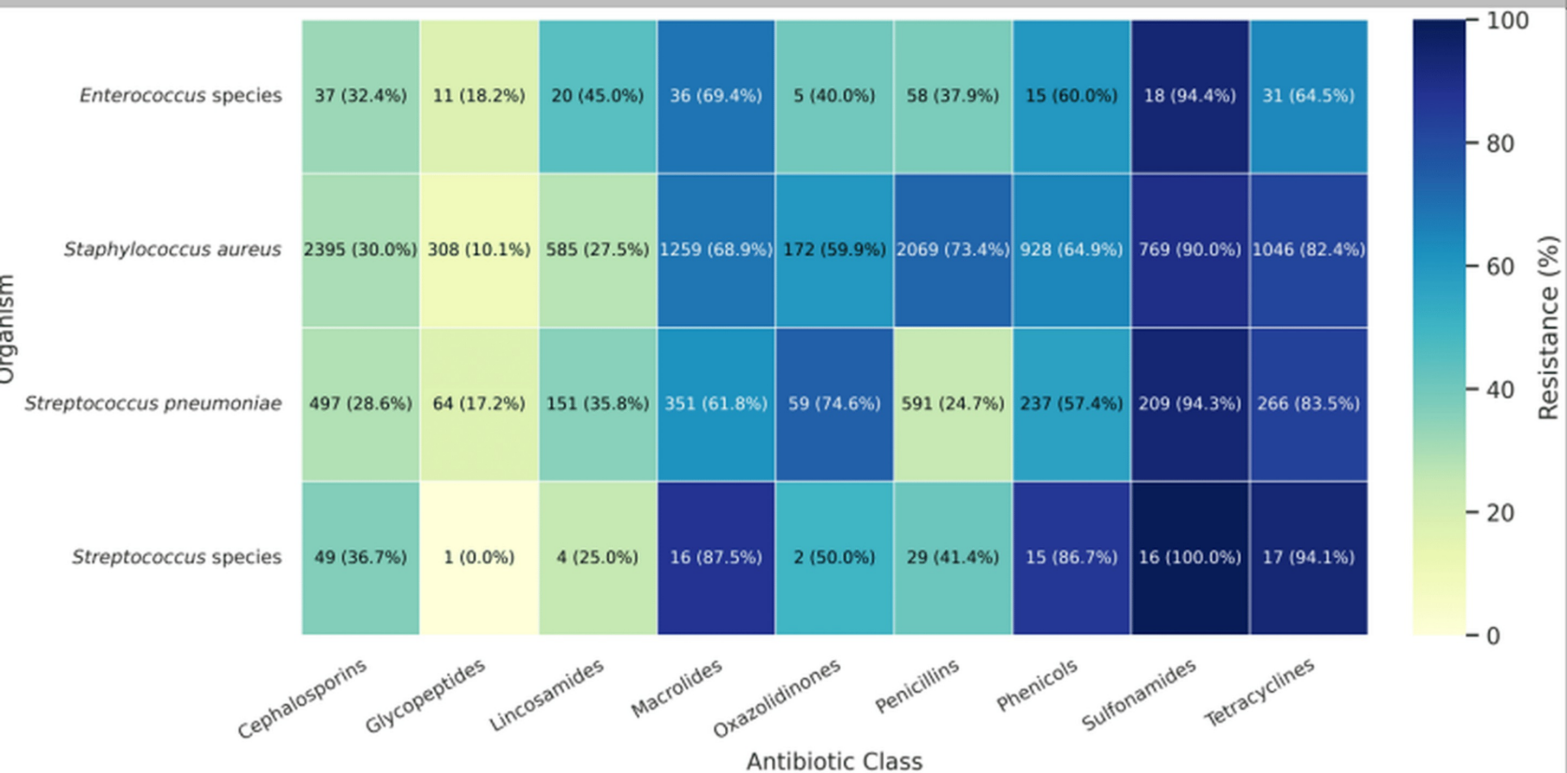
Antibiotic resistance trends in Gram-positive bacterial isolates. Heatmap showing the number of isolates tested (N) and the proportion resistant (p%) to each antibiotic class.

Among Gram-positive bacteria, high resistance to sulfonamides in *Streptococcus pneumoniae* (197/209, 94.3%) and *Staphylococcus aureus* (692/769, 90.0%) was observed. By contrast, resistance to glycopeptides was low, with *S. aureus *at 31/308 (10.1%) and *S. pneumoniae* at 11/64 (17.2%) (Figure 3).

**Figure 4:**
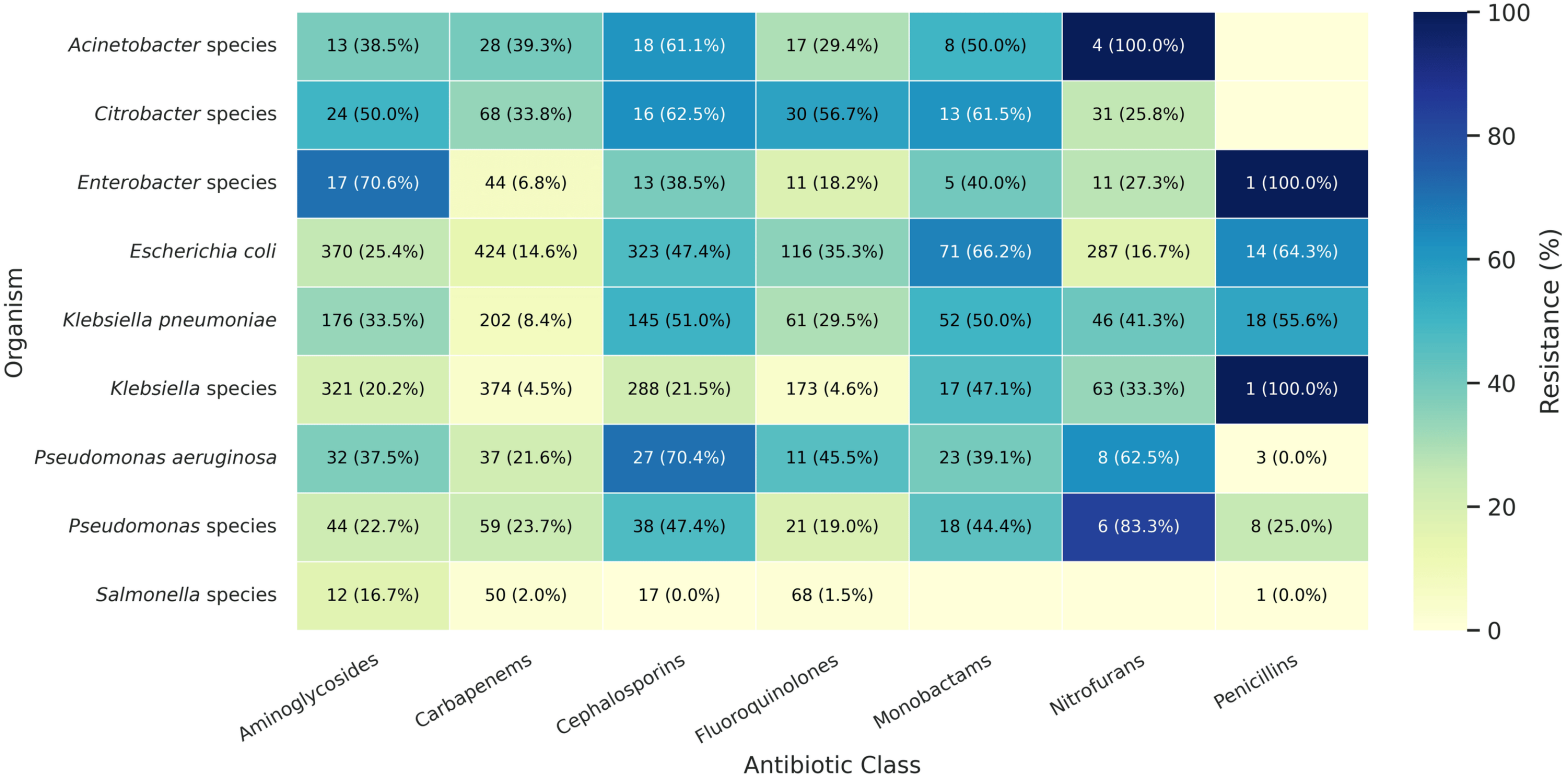
Antimicrobial resistance in Gram-negative bacteria Heatmap showing the number of isolates tested (N) and the proportion resistant (p%) to each antibiotic class.

Among Gram-negative bacteria, carbapenem resistance was high in *Acinetobacter* species (11/28, 39.3%) and *Citrobacter* spp. (23/68, 33.8%). Fluoroquinolone resistance was also notable in *Acinetobacter *species (17/30, 56.7%), *Escherichia coli *(41/116, 35.3%), and *Pseudomonas* species. The prevalence of aminoglycoside resistance was high among *Klebsiella pneumoniae* (59/176, 33.5%), *E. coli* (94/370, 25.4%), and *Enterobacter* species (12/17,70.6%) (Figure 4).

Gender-specific trends in antibiotic resistance

**Figure 5:**
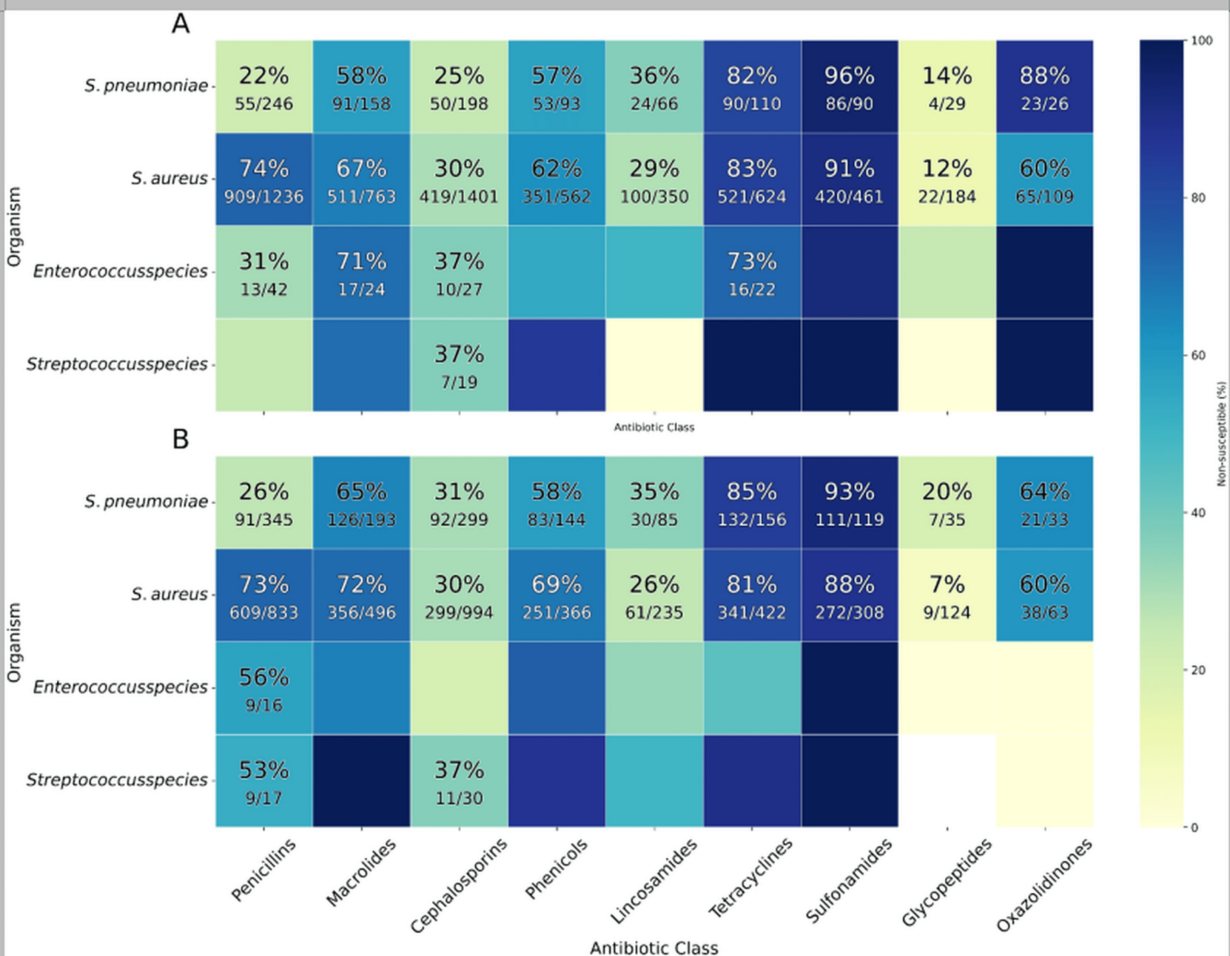
Resistance of Gram-positive isolates to antibiotic classes; stratified by patient sex. Heatmaps show the percentage of bacterial isolates that were resistant to at least one antibiotic within each class. Panel A shows isolates from female patients. Panel B shows isolates from male patients. Rows represent bacterial species and columns represent antibiotic classes. Darker shading indicates higher percentages of resistance

Antimicrobial resistance patterns showed broadly similar prevalence between sexes, with only modest variation by organism. In Gram-positive bacteria, *S. pneumoniae* and *S. aureus* demonstrated consistently high resistance across classes, while *Enterococcus* and other *Streptococcus *species showed moderate resistance with little sex difference (Figure 5).

Gram-negative bacteria

Among Gram-negative bacteria, *E. coli *and *Citrobacter* displayed the most pronounced sex variation. Male isolates of *E. coli *were more resistant to cephalosporins and fluoroquinolones, whereas female isolates of *Citrobacter* showed markedly higher carbapenem resistance. Resistance in *K. pneumoniae, P. aeruginosa,* and *Acinetobacter* species was generally comparable across sexes.

**Figure 6:**
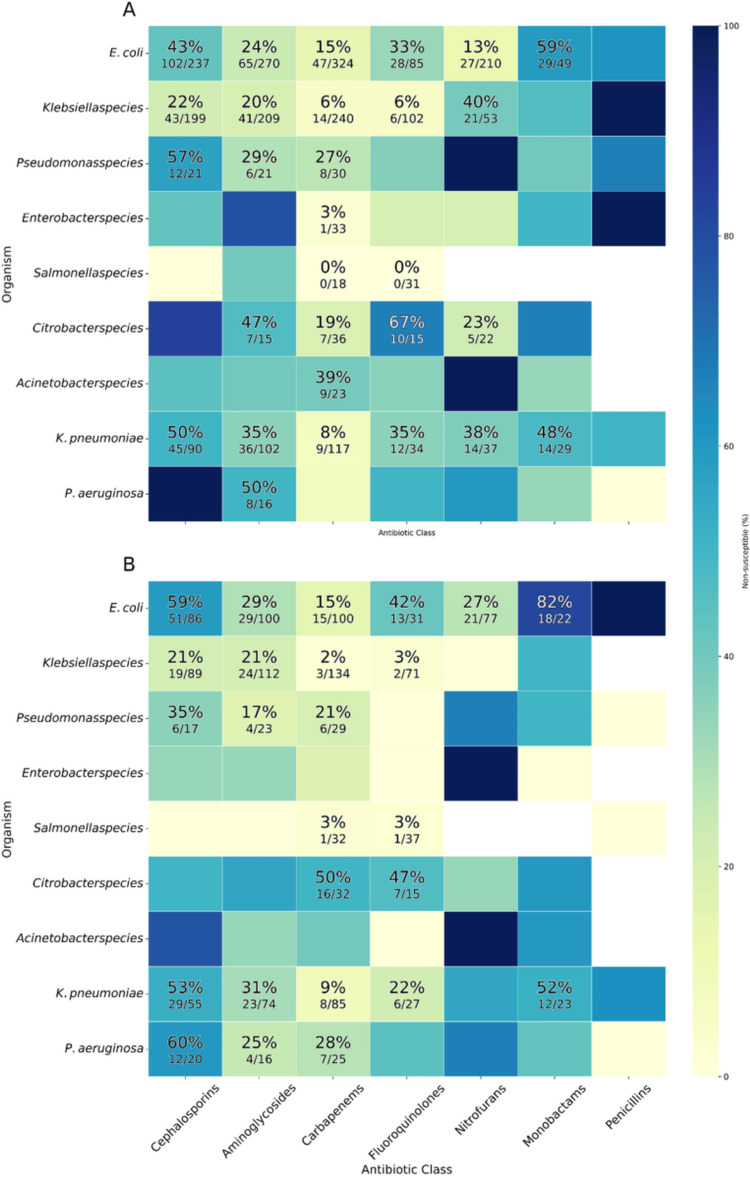
Resistance of Gram-negative isolates to antibiotic classes, shown as percentage resistant and stratified by sex. Heatmaps show the percentage of bacterial isolates resistant to at least one antibiotic within each class. Panel A shows isolates from female patients. Panel B shows isolates from male patients. Rows represent bacterial species and columns represent antibiotic classes. Darker shading indicates higher percentages of resistance

To assess whether these organism-level patterns reflected systematic sex-related differences across all strata, we compared paired female-male unadjusted prevalence estimates and modeled the odds of resistance by age and specimen type (urine and non-urine). Within matched organism-antibiotic strata, female and male groups had overall similar unadjusted prevalence of resistance. For Gram-positive bacteria, the median prevalence was 59.63% for bacteria isolated and grown from specimens obtained from female patients and 57.64% from male patients (W = 297.0, p = 0.993). For Gram-negative bacteria, medians were 38.48% and 33.33% (W = 642.0, p = 0.515), and the total prevalence was 40.00% and 41.94% (W = 1798.0, p = 0.623). Thus, when combined by age groups and specimen type, the unadjusted prevalence of resistance was generally equivalent across sexes.

However, despite broadly similar prevalence, the hospital population showed an uneven distribution of resistant isolates with an uneven ratio of resistant isolates. Female-patient specimens had a combined median of 23.08% resistant isolates compared with male-patient specimens that had 12.50% (Wilcoxon W = 991.0, p = 0.001; median difference = 6.67 percentage points). By stratum-wise medians, these were 25.00% and 13.07% (p = 0.001). In addition, the difference was larger for Gram-negative organisms (22.22% vs 9.68%; W = 295.0, p = 0.001) and smaller for Gram-positive organisms (34.42% vs 22.22%; W = 200.0, p = 0.095).

Binomial GLMs, estimating adjusted prevalence of resistance by each sex (adjusted for age band, specimen type, and interaction), showed marked epidemiological variation. Among urine specimens, women 15-49 years old had higher adjusted odds of resistance (OR = 1.063, 95% CI 1.031-1.096; p = 0.001), while among those ≥50 years, no sex difference was seen (OR = 0.973, 0.923-1.025; p = 0.301; sex × age p = 0.004).

For non-urine samples, isolates from females 15-49 years old again had larger odds (OR = 1.153, 95% CI 1.116-1.192; p < 0.001). By contrast, isolates from girls under 15 years (OR = 0.931, 0.870-0.997; p = 0.039) and from females of 50 years and above (OR = 0.884, 0.835-0.936; p = 0.003) had smaller odds, indicating comparatively greater resistance in male samples in pediatric and elderly-adult non-urine specimens. 

For the urine strata (N = 150), McFadden's pseudo-R² was 0.82 and Tjur's R² 0.30; the Brier score was 0.0137 (skill 0.85 relative to a null model). For the non-urine (N = 254), McFadden's pseudo-R² was 0.69, Tjur's R² 0.24, and the Brier score 0.0241 (skill 0.71). Pearson χ²/df was 0.07 (urine) and 0.12 (non-urine), indicating underdispersion consistent with stable grouped proportions; robust (HC1) standard errors were therefore maintained. Generalization performance was evaluated by fivefold cross-validation at the stratum level. Discrimination remained strong for held-out folds (Tjur's R² 0.28 for urine; 0.24 for non-urine). Calibration was good (Brier 0.022 and 0.035; skill 0.76 and 0.62 relative to a null model). Calibration slopes were 0.89 (urine) and 0.90 (non-urine) with intercepts 0.04 and −0.03, indicating slight over-confidence but minimal systematic bias.

Collectively, these findings suggest that while unadjusted prevalence was similar by sex, proportionately more resistant isolates occurred among female-patient specimens, largely as a result of higher adjusted prevalence in reproductive-aged women (15-49 years, particularly in urine). Isolates from men greater than 50 years, conversely, contributed disproportionately to resistance in non-urine samples. 

Patterns of MDR among bacterial isolates by sex

Of the 4,170 bacterial isolates analyzed, 2,986 (71.6%) exhibited MDR, including 36.7% classified as MDR, 29.0% as extensively drug-resistant (XDR), and 5.8% as pan-drug-resistant (PDR). Culture isolates from female patients' specimens made up a larger percentage of MDR-classified isolates (n = 1,569; 52.6%) than those received from male patients (n = 1,417; 47.4%).

*E. coli, K. pneumoniae, Klebsiella *species, and *S. pneumoniae* accounted for the majority of MDR isolates recovered from female-associated specimens. Specifically, 70.2% of MDR* E. coli* isolates, 64.3% of *K. pneumoniae*, 64.5% of *Klebsiella *spp., and 54.4% of *S. pneumoniae* MDR isolates were associated with female patients. By contrast, MDR isolates of *S. aureus* (54.1%), *P. aeruginosa* (56.4%), and *Pseudomonas* species (53.0%) were more often derived from specimens collected from male patients, particularly from non-urine sources such as blood, wound, and respiratory sample sites, typically associated with inpatient and hospital-acquired infections.

**Figure 7:**
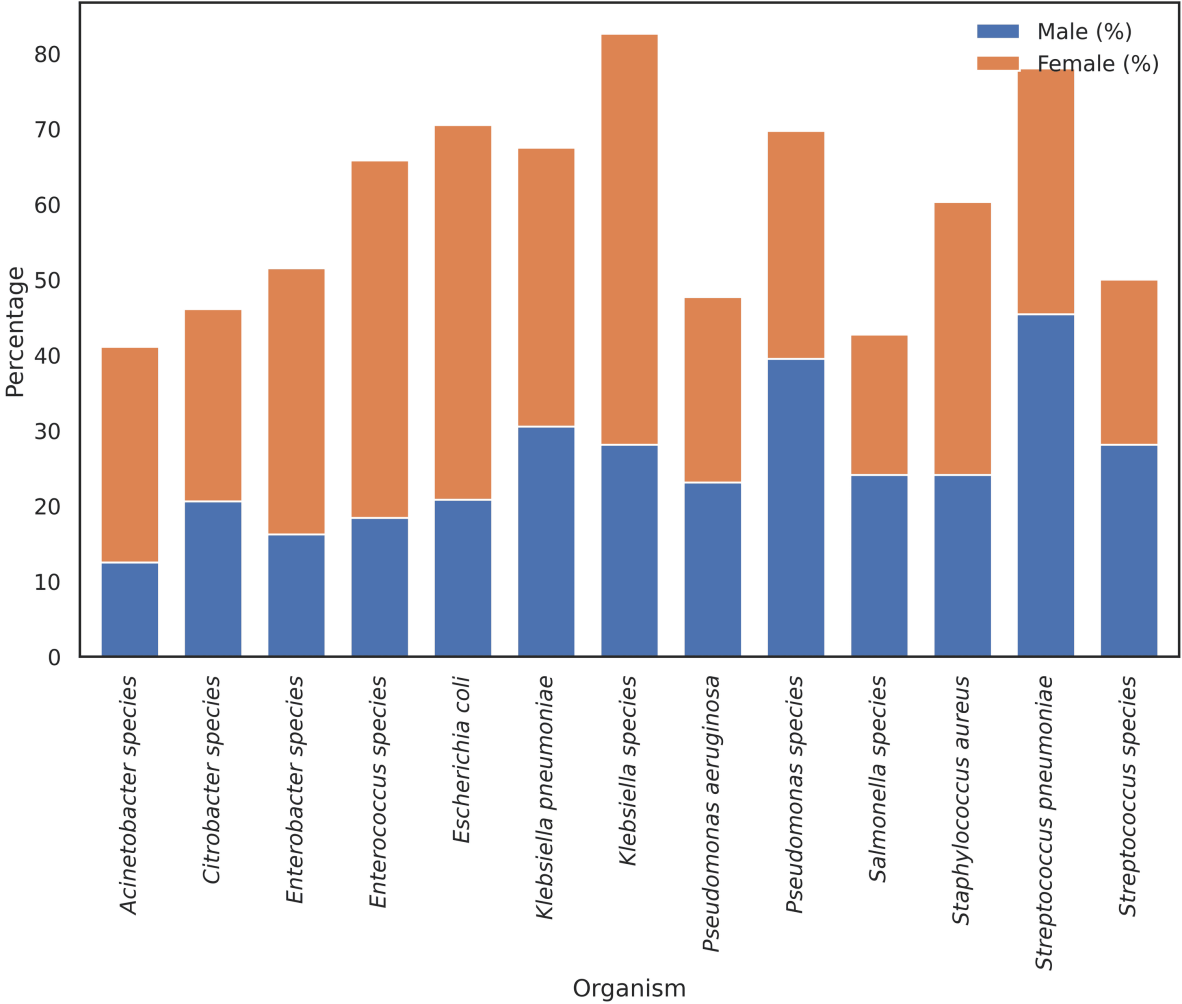
Sex distribution of multidrug-resistant (MDR) isolates Stacked bar chart displaying the percentage of female- and male-derived isolates classified as MDR for each organism.

Descriptive organism-level MDR prevalence, calculated within sex, also differed by sex. For instance, among the* E. coli* isolates, 85.2% (161/189) of male-associated isolates were MDR, whereas 73.2% (380/519) of female-associated isolates were MDR. Similarly, *K. pneumoniae *isolates were MDR in 78.4% (116/148) of male-derived and 70.9% (144/203) of female-derived isolates. On the other hand, *S. aureus* isolates were also more probable to be MDR if they were from female specimens (70.0%, 647/924) rather than male specimens (62.9%, 440/699). Patterns were similar for other organisms except for a more equal distribution by sex or heterogeneity by sample setting and size. *Citrobacter* spp., *Acinetobacter *spp., and *Enterobacter* spp. had high overall rates of MDR prevalence in both sexes (Figure 7). 

Adjusted associations between sex and MDR: multivariable modeling

Using a binomial generalized linear model (GLM), the adjusted odds of MDR were estimated across strata defined by organism, specimen type, age group, and care setting. Across the dataset, isolates from specimens collected from male patients were more often MDR. In the multivariable model, the female vs. male odds of MDR for all isolates combined were 0.35 (95% CI 0.20-0.63), indicating that isolates linked to female patients had approximately 65% lower odds of MDR. Independent of sex, isolates from outpatient care had higher odds of MDR than those from inpatient care (aOR 1.37, 1.30-1.44).

The clearest sex difference was observed in urinary isolates, which accounted for the largest volume of data. Urine collected from the Outpatient Department of female patients had dominant uropathogens that included *E. coli *(aOR 0.35, 0.32-0.37), *K. pneumoniae* (aOR 0.77, 0.72-0.81), and *Klebsiella* spp. (aOR 0.76, 0.71-0.80) with low cases of MDR. While urine samples from female patients in the Inpatient Department, *E. coli* showed a similar pattern (aOR 0.52, 0.48-0.57).

Several important non-urine strata also showed lower MDR among isolates from female-linked specimens. For *S. pneumoniae *in inpatients, the aOR was 0.49 (0.44-0.55). For *Pseudomonas* spp. in outpatient specimens, the aOR was 0.54 (0.43-0.68), and for *P. aeruginosa* in outpatient specimens, the aOR was 0.71 (0.50-0.99). A notable null finding was *E. coli* in outpatient non-urine specimens (aOR 0.91, 0.70-1.20; p = 0.507). By contrast, higher MDR among isolates from female-linked specimens was concentrated in non-urine *S. aureus* inpatient non-urine OR 1.83 (1.69-1.98) and outpatient non-urine aOR 1.57 (1.50-1.65), whereas *S. aureus* in outpatient urine showed lower odds in female-linked isolates (aOR 0.86, 0.85-0.88), highlighting specimen-specific heterogeneity.

Age modified the observed sex differences in resistance. Among adults aged 15-49 years,* E. coli *isolates showed lower resistance in females compared with males, both in outpatient urine (OR 0.42) and inpatient non-urine specimens (OR 0.47). In adults ≥50 years, *E. coli *resistance in outpatient urine remained lower in females (OR 0.36), but the pattern reversed for *Klebsiella* spp. in outpatient non-urine specimens, where resistance was higher in female-linked isolates (OR 1.99). In children (<15 years), the direction of sex differences varied by organism and specimen: for example, *S. aureus* in inpatient non-urine (OR 1.36) and *E. coli* in outpatient non-urine (OR 1.42) were higher in female-linked isolates, whereas for *Klebsiella* spp. (OR 0.58) and *Salmonella* spp. (OR 0.70) in outpatient, non-urine specimens were lower.

Overall, the results demonstrate that male patients carry a disproportionate burden of MDR in most urinary and respiratory infections, particularly in reproductive-aged adults, while female patients face elevated risk in specific non-urinary contexts, most notably *S. aureus* and some Enterobacterales.

Model performance

The multivariable model had good overall performance. McFadden's pseudo-R² was 0.71, and Tjur's R² was 0.28, indicating high explanatory power. Discriminatory performance was stable over fivefold cross-validation, with Tjur's R² ranging from 0.26 to 0.29. The Brier score was 0.019, equivalent to a skill score of 0.78 against a null model. Calibration measures were satisfactory, with a slope of 0.92 and an intercept of 0.01. Residual dispersion was low (Pearson χ²/df = 0.09).

## Discussion

The findings of this study highlight the critical role of gender in shaping antimicrobial resistance patterns in Sub-Saharan Africa. With over 92% of bacterial isolates exhibiting resistance to at least one antibiotic and 65% meeting the definition of MDR, the data reflect a heavy burden of AMR at Mbarara Regional Referral Hospital. More importantly, the analysis uncovers pronounced and statistically significant gender disparities, with female-derived isolates exhibiting consistently higher resistance rates across key antibiotic classes, particularly beta-lactams, fluoroquinolones, and aminoglycosides.

These disparities align with prior research indicating that women are frequently exposed to broad-spectrum antibiotics, particularly in the management of recurrent urinary tract infections and reproductive health conditions, including bacterial vaginosis, pelvic inflammatory disease, and post-surgical prophylaxis [13,14].

UTIs are among the most common bacterial infections globally, and women have a higher predisposition due to anatomical factors such as a shorter urethra, which facilitates bacterial entry into the bladder [15]. In addition, pregnancy and the use of contraceptives have been associated with an increased risk of UTIs in women [16-18]. Pregnancy induces physiological changes, including hormonal shifts and urinary stasis due to uterine pressure on the bladder, which predisposes expectant mothers to UTIs [18]. Similarly, contraceptives can alter vaginal and urinary tract microbiota, reducing protective normal flora such as lactobacilli and facilitating bacterial colonization, thereby increasing the risk of UTIs [17,19].

In LMICs like Uganda, where diagnostic capacity is often limited, the frequent recurrence of UTIs among women is also more likely to be prescribed antibiotics empirically, amplifying selective pressure and resistance, particularly *E. coli*, the primary UTI pathogen [7,20]. In addition, *S. aureus* is frequently implicated in skin and soft tissue infections, potentially linked to antibiotic use for reproductive and postnatal infections among women [21,22].

MDR exhibited distinctive, clinically meaningful sex patterns after controlling for organism and specimen. In the adjusted multivariable model for age group, organism, specimen, setting of care, and their interactions, isolates from specimens from female patients had decreased overall odds of MDR (aOR 0.35, 95% CI 0.20-0.63). This summary effect was largely driven by the urinary strata, where male-submitted *E. coli* and *Klebsiella *sp. urine isolates were more often MDR in both inpatient and outpatient care, aligning with reported higher MDR among male urinary *E. coli*. Male predominance of urinary MDR likely reflects urologic comorbidities and interventions in men, including obstructive uropathy, prostatitis, urinary stones, urologic malignancy, and chronic catheter or stent use, which promote biofilm persistence and recurrent bacteriuria, which increases cumulative exposure to broad-spectrum antibiotics, thereby selecting for resistant strains [20].

*S. aureus* from non-urine samples had higher MDR among female-associated isolates of inpatients and outpatients, as per gendered care roles and exposure in peri-operative and obstetric-gynecologic services, where wound care, device usage, and empiric prophylaxis increase selection pressure. This supports the hypothesis that antibiotic exposure patterns play a critical role in resistance development, thereby echoing global findings on the feminization of AMR burden in community-acquired infections [5,23]

Conversely, higher MDR prevalence in male-derived *P. aeruginosa* isolates likely reflects gendered exposure to nosocomial pathogens, as men are often more represented in ICU admissions and invasive procedures associated with healthcare-acquired infections [24,25]. In addition, men are more likely to only present themselves in hospital with chronic conditions such as diabetes, chronic obstructive pulmonary disease (COPD), and other immunosuppressive diseases [26], which usually require prolonged hospitalization and long antibiotic regimens, further increasing the risk of MDR development. Men also exhibit higher rates of smoking and alcohol consumption, which have been associated with impaired immune function and an increased risk of hospital-acquired infections [26]. These lifestyle factors, combined with higher rates of occupational exposure to environmental pathogens in industries such as construction, mining, and agriculture, may contribute to increased AMR burden in male patients [26,27]. Furthermore, adherence to treatment regimens tends to be lower among men, potentially leading to the selection of resistant bacterial populations [28,29]. These differences emphasize that AMR is not only a microbiological issue but also a sociostructural one, shaped by gendered access to care, prescribing patterns, and infection risks.

The World Health Organization’s Global Antimicrobial Resistance Surveillance System (GLASS) encourages the collection of sex-disaggregated data [30], yet implementation remains uneven in many LMICs. In addition, public health messaging and stewardship campaigns should consider gender in their design and delivery. For example, embedding AMR education in reproductive health services and antenatal clinics could help reduce inappropriate antibiotic use among women. At the same time, improving hospital infection prevention for male patients, particularly in high-risk units, may reduce exposure to resistant nosocomial organisms.

As a retrospective study, it relies on existing hospital records, which may be subject to missing data. Additionally, the lack of molecular characterization of resistance mechanisms limits deeper insights into the genetic determinants of AMR disparities. Future research should explore the molecular mechanisms underlying observed gender differences, the role of self-medication, and intersectional factors such as age, socioeconomic status, and comorbidities contributing to gender-based AMR trends. Understanding these dynamics can inform targeted interventions that improve both clinical outcomes and health system equity.

## Conclusions

Integrating a gender lens into antimicrobial resistance research is essential for guiding equitable clinical and public health action. Current surveillance in Uganda does not capture any gender-related factors such as pregnancy status, contraceptive use, healthcare access, self-medication, and caregiving roles, yet these may strongly influence patterns of antimicrobial exposure and resistance. Addressing this gap will require collecting data that captures gender-related factors more directly, so that we can better explain why resistance disparities occur. In addition, designing AMR control strategies that are fair, practical, and responsive to the needs of communities, especially in low-resource settings like Uganda.

## References

[1] (2022). Global burden of bacterial antimicrobial resistance in 2019: a systematic analysis. Lancet.

[2] Muhwezi I, Bazira J, Zamarano H (2022). Quantification and molecular characterization of extended spectrum beta-lactamase producing enterobacteriaceae from agropastoral communities of Mbarara district, south western Uganda. Res Sq.

[3] Mayito J, Kibombo D, Olaro C (2024). Characterization of antibiotic resistance in select tertiary hospitals in Uganda: an evaluation of 2020 to 2023 routine surveillance data. Trop Med Infect Dis.

[4] Tacconelli E, Sifakis F, Harbarth S (2018). Surveillance for control of antimicrobial resistance. Lancet Infect Dis.

[5] Walkty A, Karlowsky JA, Lagace-Wiens P, Baxter MR, Adam HJ, Zhanel GG (2022). Antimicrobial resistance patterns of bacterial pathogens recovered from the urine of patients at Canadian hospitals from 2009 to 2020. JAC Antimicrob Resist.

[6] Gautron JM, Tu Thanh G, Barasa V, Voltolina G (2023). Using intersectionality to study gender and antimicrobial resistance in low- and middle-income countries. Health Policy Plan.

[7] Lynch I, Fluks L, Manderson L (2024). Gender and equity considerations in AMR research: a systematic scoping review. Monash Bioeth Rev.

[8] Waterlow NR, Ashfield T, Knight GM (2025). Observational study of antibiotic prescribing patterns by age and sex in primary care in England: why we need to take this variation into account to evaluate antibiotic stewardship and predict AMR variation. JAC Antimicrob Resist.

[9] Longes DF, Tibaijuka L, Muwanguzi M (2025). Self-reported quality of life and lived experiences of adolescent cancer survivors aged 10-19 in southwestern Uganda: a mixed-methods study in a resource-limited setting. Cancer Rep (Hoboken).

[10] Cheesbrough M (2005). District laboratory practice in tropical countries part 2.

[11] Tang KW, Millar BC, Moore JE (2023). Antimicrobial resistance (AMR). Br J Biomed Sci.

[12] Rafailidis PI, Kofteridis D (2022). Proposed amendments regarding the definitions of multidrug-resistant and extensively drug-resistant bacteria. Expert Rev Anti Infect Ther.

[13] Silva A, Costa E, Freitas A, Almeida A (2022). Revisiting the frequency and antimicrobial resistance patterns of bacteria implicated in community urinary tract infections. Antibiotics (Basel).

[14] Makeri D, Dilli PP, Nyaketcho D (2023). Prevalence of urinary tract infections in Uganda: a systematic review and meta-analysis. Open Access Lib J.

[15] Izett-Kay M, Barker KL, McNiven A, Toye F (2022). Experiences of urinary tract infection: a systematic review and meta-ethnography. Neurourol Urodyn.

[16] Warzecha D, Pietrzak B, Urban A, Wielgoś M (2021). How to avoid drug resistance during treatment and prevention of urinary tract infections. Prz Menopauzalny.

[17] Heidemann J, Kalder M, Kostev K (2022). Association between contraceptive use and risk of lower urinary tract infection (LUTI): a case-control study. Int J Clin Pharmacol Ther.

[18] Patnool RB, Wadhwani A, Balasubramaniam V (2022). Antimicrobial resistance and implications: impact on pregnant women with urinary tract infections. J Pure Appl Microbiol.

[19] Lo C, Abraham A, Bejan CA, Reasoner SA, Davidson M, Lipworth L, Aronoff DM (2023). Contraceptive exposure associates with urinary tract infection risk in a cohort of reproductive-age women: a case control study. Eur J Contracept Reprod Health Care.

[20] Endale H, Mathewos M, Abdeta D (2023). Potential causes of spread of antimicrobial resistance and preventive measures in one health perspective-a review. Infect Drug Resist.

[21] Johnson B, Stephen BM, Joseph N, Asiphas O, Musa K, Taseera K (2021). Prevalence and bacteriology of culture-positive urinary tract infection among pregnant women with suspected urinary tract infection at Mbarara regional referral hospital, South-Western Uganda. BMC Pregnancy Childbirth.

[22] Nakawuki AW, Nekaka R, Ssenyonga LV, Masifa G, Nuwasiima D, Nteziyaremye J, Iramiot JS (2022). Bacterial colonization, species diversity and antimicrobial susceptibility patterns of indwelling urinary catheters from postpartum mothers attending a tertiary hospital in Eastern Uganda. PLoS One.

[23] Keenan K, Silva Corrêa J, Sringernyuang L, Nayiga S, Chandler CI (2025). The social burden of antimicrobial resistance: what is it, how can we measure it, and why does it matter?. JAC Antimicrob Resist.

[24] Baccolini V, Migliara G, Isonne C (2021). The impact of the COVID-19 pandemic on healthcare-associated infections in intensive care unit patients: a retrospective cohort study. Antimicrob Resist Infect Control.

[25] Ahmat AM, Bolti MA, Yandai FH (2023). Antimicrobial resistance of Pseudomonas aeruginosa isolated from human infections in N’Djamena, Chad. Open J Med Microbiol.

[26] Dias SP, Brouwer MC, van de Beek D (2022). Sex and gender differences in bacterial infections. Infect Immun.

[27] Bugeza JK, Roesel K, Mugizi DR (2024). Sero-prevalence and risk factors associated with occurrence of anti-Brucella antibodies among slaughterhouse workers in Uganda. PLoS Negl Trop Dis.

[28] Zarauz JM, Zafrilla P, Ballester P, Cerda B (2022). Study of the drivers of inappropriate use of antibiotics in community pharmacy: request for antibiotics without a prescription, degree of adherence to treatment and correct recycling of leftover treatment. Infect Drug Resist.

[29] Kiggundu R, Wittenauer R, Waswa JP (2022). Point prevalence survey of antibiotic use across 13 hospitals in Uganda. Antibiotics (Basel).

[30] Waterlow NR, Cooper BS, Robotham JV, Knight GM (2024). Antimicrobial resistance prevalence in bloodstream infection in 29 European countries by age and sex: an observational study. PLoS Med.

